# A prognostic score for overall survival in patients treated with abiraterone in the pre- and post-chemotherapy setting

**DOI:** 10.18632/oncotarget.27133

**Published:** 2019-08-20

**Authors:** Martin Boegemann, Katrin Schlack, Lena Früchtenicht, Julie Steinestel, Andres Jan Schrader, Yvonne Wennmann, Laura-Maria Krabbe, Okyaz Eminaga

**Affiliations:** ^1^ Department of Urology, University of Muenster Medical Center, Muenster, Germany; ^2^ Department of Urology, Augsburg Medical Center, Augburg, Germany; ^3^ Department of Urology, University of Texas Southwestern Medical Center, Dallas, TX, USA; ^4^ Department of Urology, Stanford Medical School, Stanford, CA, USA

**Keywords:** risk score, mCRPC, abiraterone, survival, outcome

## Abstract

**Background:** Therapy resistance remains a serious dilemma in metastatic castration-resistant prostate cancer (mCRPC) with primary or secondary resistance frequently occurring against any given therapy. Available prognostic models for Abiraterone Acetate (AA) are specifically designed for either pre- or post-chemotherapy settings and mostly based on trial datasets not necessarily reflecting real-life.

**Results:** A score of 0–2 (low-risk) is associated with an OS-probability of 80.0% (95%CI: 71.3–90.6) and 50.5% (95%CI: 38.7–66.0) after 1 and 2 years while a score of 3–4 (high risk) is associated with an OS-probability of 35.3% (95%CI: 22.3–55.8) and 5.7% (95%CI: 1.5–21.8), respectively. The bootstrapping survival analysis of the scoring-system revealed a median c-index of 0.80 (IQR: 0.79–0.82).

**Material and Methods:** We developed a scoring-system using four real-life parameters 117 mCRPC patients treated with AA either pre- or post-chemotherapy. These parameters were evaluated using COX regression analysis. The scoring-system consists of binary-categorized parameters; when any of these exceeds the given cut-off, one point is added up to a final score ranging between 0–4 points. The final score was stratified by a median threshold of 2 into low- and high-risk groups. We evaluated the discriminative ability of our scoring-system using concordance probability (C-index) and Kaplan–Meier-analysis and applied a 100-times bootstrap for survival analysis.

**Conclusions:** Our study introduces a novel prognostic scoring-system for OS of real-life mCRPC patients receiving AA treatment irrespective of the line of therapy. The scoring-system is simple and can be easily utilized based on PSA and LDH values, neutrophil to lymphocyte ratio, and ECOG performance status.

## INTRODUCTION

Prostate cancer (PCa) is the most common cancer in males [1]. Although the majority of cases is showing a favorable course of disease, PCa still remains the 2nd most common cause of cancer related death worldwide [1]. Most men will die in the state of metastatic castration resistant prostate cancer (mCRPC), the most severe stage of PCa development. In recent years, several treatment compounds have become available for mCRPC [2–10]. However, therapy resistance remains a serious dilemma in mCRPC treatment since primary or secondary resistance frequently occurs against any given therapy. This complicated situation challenges the physicians treating mCRPC patients and affords additional tools to reach a consensus about the personalized treatment plan.

Abiraterone acetate (AA), in combination with prednisone, is one of the drugs playing a pivotal role in mCRPC treatment. AA is approved for the treatment of asymptomatic or oligosymptomatic mCRPC-patients prior or after taxane-based chemotherapy. In addition, several biomarkers have been proposed as tools for personalized treatment plans. For example, testing for circulating AR-v7 splice variants has shown to help to identify patients with high risk for AA resistance [11, 12]. However, AR-v7 testing is expensive and not commonly available. Other baseline biomarkers like PSA (prostate-specific antigen), LDH (lactic acid dehydrogenase), AP (alkaline phosphatase), neutrophil to lymphocyte ratio (NLR), or pain-intensity have been identified to improve selecting patients with superior benefit from AA treatment [13–15]. The major advantage of these baseline biomarkers is their wide availability and their utilization in clinical routine. However, these biomarkers are limited by their moderate accuracy. To strengthen the predictive accuracy of these biomarkers, several prognostic scores combining biomarkers in the context of AA treatment have been introduced [16–18]. Nevertheless, the available prognostic models within AA therapy are specifically designed for either pre- or post-chemotherapy patients and are mostly based on datasets from randomized controlled trials (RCTs) which do not mandatorily reflect clinical routine. In this study we introduce a novel prognostic scoring system for patients receiving AA within the pre- or post-chemotherapy setting. Further, we evaluate this prognostic score on a dataset originating from clinical routine.

## RESULTS

**Figure 1:**
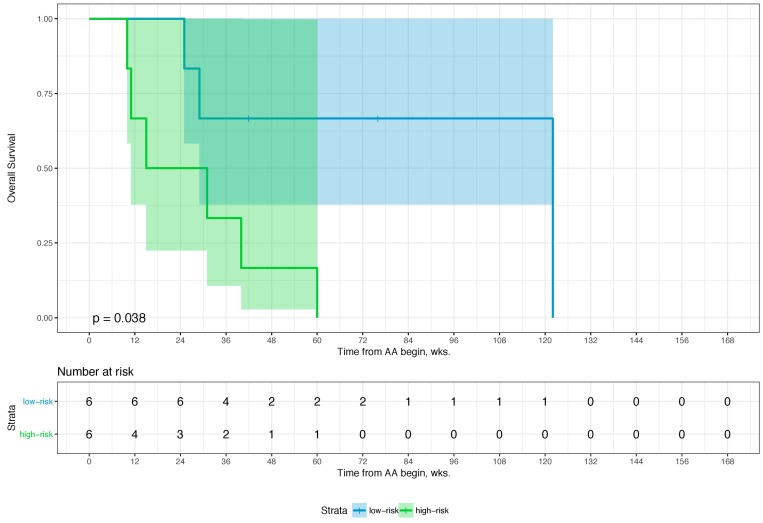
Kaplan–Meier Curve of a balanced test set (n = 12) from the first run of 100-times bootstrap resampling. Log-rank was given. The colored area represents the 95% confidence interval.

**Figure 2:**
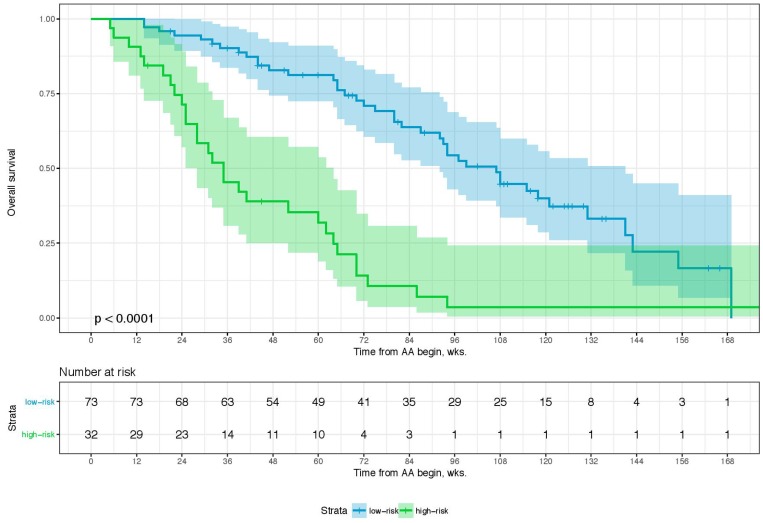
Kaplan–Meier Curve… of the remaining data set (*n* = 105) from the first run of 100-times bootstrap resampling. Log-rank was given. The colored area represents the 95% confidence interval.

Sixty-eight percent (*n* = 80) of the patients died during a median follow-up of 62.0 weeks (wks.) (IQR: 32–99 wks.). Overall, the 1-year and 2-year overall survival (OS) rates were 66.9% (95% CI: 57.7–77.6) and 39.4% (95% CI: 29.8–52.0), respectively. Table 1 shows an overview of the relevant clinicopathological characteristics of the study cohort. The *p*-values given demonstrate the degree of deviation of binomial distribution with a value of >0.05 relating to a distribution not being influenced by selection bias. A combination of four parameters is associated with overall survival (i.e., Eastern Collaborative Oncology Group performance status (ECOG), PSA- and LDH-level, and the NLR). The optimal cutoffs for PSA, LDH, and NRL is 58 ng/ml, 229 U/L, and 3.7, respectively (Table 2). Table 3 shows the univariate and multivariate analyses of these parameters for OS, which comprises our scoring System for the prognostication of OS after 1 and 2 yrs. The score sum then allows to group the patients into two prognostic categories for 1-year and 2-year OS (i.e., low- and high-risk). A score of 0–2 (low-risk) is associated with an OS-probability of 80.0 (95%CI: 71.3–90.6) and 50.5% (95%CI: 38.7–66.0) after 1 and 2 years while a score of 3–4 (high risk) is associated with an OS-probability of 35.3 (95%CI: 22.3–55.8) and 5.7% (95%CI: 1.5–21.8), respectively (Table 4). The distribution of the risk score of 0–2 points and 3–4 points differed significantly in-between patients in both the pre- and the post-chemotherapy setting (Table 5). When we stratified these risk groups by chemotherapy status, we observed that chemotherapy-naïve patients with low-risk scores (OS-probability of 89%) have significantly more survival benefit from AA compared to those with high-risk scores who were chemotherapy-naïve as well (OS-probability of 23.8%). Further, chemotherapy-naïve patients with low-risk scores have a superior survival outcome in comparison to low-risk patients who already received chemotherapy prior to AA. The chemotherapy status was not significantly relevant for survival outcome in patients with high-risk scores (Table 4). Furthermore, median treatment times were almost identical for 0–2 and 3–4 risk-factors, regardless of chemotherapy status (Table 5) indicating the importance of these risk factors towards duration of benefit for both pre- and-post chemotherapy setting of AA-therapy (58 and 54.6 wks. for 0–2 risk-factors vs. 32.2 and 33.1 wks. for 3–4 risk-factors, respectively) as well as survival. The bootstrapping survival analysis of the score system revealed a median c-Index of 0.80 (interquartile range (IQR): 0.79–0.82). Here, we randomly chose 6 low- and 6 high-risk patients out of our cohort each time as a test set for KM analysis (Supplementary Figure 1). Figure 1 shows a Kaplan–Meier curve of the test set, whereas Figure 2 includes the remaining subset with 105 patients that were excluded in the first run of the bootstrapping analysis. For the whole cohort (*n* = 117), characteristics subdivided in the low- and high-risk groups are given in Table 5. The median OS here was 99 wks. (95%CI: 92–131) and 35 wks. (95%CI: 28–60), respectively. The power and the effect size of the score system with respect to the overall survival was 0.9999 and 0.798, respectively.

**Table 1:** The characteristics of the study cohort

Variable	all	*p*
Patients [*n*], (%)	117 (100%)	-
Age median [years] (IQR)	70.0 (64–76.0)	0.304^**^
Lymph-nodal metastases [*n*] (%)	74 (63.2)	0.004^**^
Visceral metastases [*n*] (%)	30 (25.6)	<0.001^**^
Bone metastases [*n*] (%)	102 (87.2)	<0.001^**^
Pre-chemotherapy [*n*] (%)	69 (56.0)	0.052^**^
Post-chemotherapy [*n*] (%)	48 (41.0)	0.052^**^
Patients died [*n*] (%)	80 (68.4)	<0.001^*^
ECOG (all) [*n*] (%)		
0–1	94 (80.3)	<0.001^*^
≥2	23 (19.7)
Gleason score ≥8 [*n*] (%)	54 (46.2)	0.4597 ^**^
Median PSA Baseline [ng/ml] (IQR)	122.0 (44.0–348.0)	0.6231^**+^
Median LDH Baseline [U/l] (IQR)	255.0 (214.0–369.0)	0.009^**^
Median NLR Baseline [Ratio] (IQR)	3.467 (2.693–4.383)	0.1308^**^
Median Ca^+2^ [U/l] (IQR)	2.250 (2.170–2.340)	0.9966^**^
Median OS Time (Weeks) (QR)	62 (32–99)	0.0937^**^

Abbreviations: IQR: Inter quartile range; ECOG: Eastern Collaborative Oncology Group; PSA: Prostate specific antigen; LDH: Lactate dehydrogenase; NLR: Neutrophil to lymphocyte ratio; OS: Overall survival. The *p*-values given demonstrate the degree of deviation of binomial distribution with a value of >0.05 relating to a distribution not being influenced by selection bias ^*^Binomial test, ^**^One-sample Kolmogorov-Smirnov test for binomial distribution (^+^After log2 transformation).

**Table 2:** Determination of sophisticated cutoffs for the continues parameters associated with overall survival using “Youden” approach

Factors	Cut off	AUC for OS (95% CI)
PSA [ng/ml]	58	0.737 (0.635–0.840)
LDH [U/l]	229	0.761 (0.667–0.854)
NLR [Ratio]	3.7	0.655 (0.552–0.757)

Abbreviations: PSA: Prostate specific antigen; LDH: Lactate dehydrogenase; NLR: Neutrophil to lymphocyte ratio; AUC: Area under the curve; OS: Overall survival.

**Table 3:** Univariate and multivariate cox regression analyses for overall survival

	Univariate	*P* value	Multivariate	*P* value
**Factor**	HR (95% Cl)	(C-index)	HR (95% CI)	(C-index)
**ECOG -Categorized-**	3.3465 (1.90–5.9)	<0.001 (0.603)	2.761 (1.53–4.99)	<0.001 (0.742+)
**PSA [ng/ml] -Categorized-**	2.225 (1.24–3.94)	0.003 (0.599)	1.774 (0.96–3.29)	
**LDH [U/l] -Categorized-**	3.130 (1.78–5.58)	<0.001 (0.662)	2.639 (1.45–4.81)	
**NLR [Ratio] -Categorized-**	1.76 (1.129–2.756)	0.01 (0.594)	1.183 (0.70–1.99)	

Abbreviations: HR: Hazard ratio; CI: Confidence interval; ECOG: Eastern Collaborative Oncology Group; PSA: Prostate specific antigen; LDH: Lactate dehydrogenase; NLR: Neutrophil to lymphocyte ratio. + *p*-value was calculated without bootstrapping and based on this cox regression analysis.

**Table 4:** The 4-parameters scoring system for the risk classification of CRCP patients to estimate the overall survival probability

Criteria	Cut off	Point	OS probability	
**ECOG**	2	1	-	
**PSA [ng/ml]**	58	1	-	
**LDH [U/l]**	229	1	-	
**NLR [Ratio]**	3.7	1	-	
**Total points**		4	-	
**Sum points** *(Log rank P = < 0.001)*			1-year (95% CI) OS survival probability	2-year (95% CI) OS survival probability
**0–2**			80.0% (71.3–90.6)	50.5% (38.7–66.0)
**3–4**			35.3% (22.3–55.8)	5.7% (1.5–21.8)
**Stratified by chemotherapy status**				
**Score: 0–2** *(Log rank P = 0.0482)*				
**Pre-chemotherapy**			89.0% (80.3–98.7)	58.0% (43.4–77.4)
**Post-chemotherapy**			63.0% (47.2–84.1)	37.0% (22.7–60.6)
**Score: 3–4** *(Log rank P = 0.4)*				
**Pre-chemotherapy**			23.8% (10.1–55.9)	n.c.
**Post-chemotherapy**			42.9% (26.2–70.2)	5% (0.8–35.8)

Abbreviations: OS: Overall survival; ECOG: Eastern Collaborative Oncology Group; PSA: Prostate specific antigen; LDH: Lactate dehydrogenase; NLR: Neutrophil to lymphocyte ratio; CI: Confidence interval. C-index of this score system is after bootstrapping 0.80 (IQR: 0.79–0.82). Log rank test was applied to determine the significance of survival differences. N.c.: Not calculable because of not reaching 2-yr. survival time.

**Table 5:** The population characteristics of patients having scores 0–2 or 3–4

Variable	0–2 points (low risk)	3–4 points (high risk)	*P* value
Patients [*n*], (%)	79 (67.5)	38 (32.5)	-
Age median [years] (IQR)	71 (68.3–72.3)	68.5 (65.6–71.4)	<0.001^*^
Lymphondal metastases [*n*] (%)	52 (65.8)	22 (57.9)	0.33^**^
Visceral metastases [*n*] (%)	17 (21.5)	13 (34.2)	0.2103^**^
Bone metastases [*n*] (%)	68 (86.1)	34 (89.5)	0.6771^**^
Pre-chemotherapy [*n*] (%)	52 (75.4)	17 (24.6)	0.03189^**^
Post-chemotherapy [*n*] (%)	27 (56.3)	21 (43.7)	0.03189^**^
AA treatment duration			
Pre-chemotherapy [Weeks], (IQR)	58 (49.8–66.2)	32.2 (17.8–46.6)	<0.001^*^
Post-chemotherapy [Weeks], (IQR)	54.6 (42.9–66.2)	33.1 (24.0–42.2)	<0.001^*^
Patients died [*n*] (%)	44 (55.0)	36 (94.7)	<0.001^**^
-Died before chemotherapy [*n*] (%)	22 (50.0)	16 (44.4)	<0.001^**^
-Died after chemotherapy [*n*] (%)	22 (50.0)	20 (55.6)	0.322^**^
ECOG (all) [*n*] (%)		
0–1	77 (81.9)	17 (18.1)	<0.001^**^
≥2	2 (8.7)	21 (91.3)	
Gleason score ≥8 [*n*] (%)	36 (45.6)	22 (57.9)	0.8535^*^
Median PSA Baseline [ng/ml] (IQR)	46.5 (29.2–181.9)	182 (258.2–463.3)	<0.001^*^
Median LDH Baseline [U/l] (IQR)	200.5 (189.1–207.5)	308 (378.5–526.0)	<0.001^*^
Median NLR Baseline [Ratio]	2.9 (2.52–3.16)	3.9 (3.7–4.5)	<0.001^*^
Median Ca^+2^ [U/l]	2.31 (2.27–2.36)	2.23 (2.19–2.25)	<0.001^*^
Median OS Time [Weeks] (IQR)	99 (92–131)	35 (28–60)	<0.001^*^

Abbreviations: IQR: Inter quartile range; ECOG: Eastern Collaborative Oncology Group; PSA: Prostate specific antigen; LDH: Lactate dehydrogenase; NLR: Neutrophil to lymphocyte ratio; OS: Overall survival; ^*^Mann-Whitney-Wilcoxon Test; ^**^Chi-Squared Test.

## DISCUSSION

Several prognostic scores have been introduced for outcome prognostication in mCRPC patients [16–23]. The most commonly used tool for survival prognosis in mCRPC is the nomogram developed by Halabi *et al*. [21]. However, their tool has followed a general approach comprising the prognosis for all mCRPC patients regardless of the given or following therapy regimes. In a domain with growing treatment options and consequently life-prolonging capacity, it is increasingly important to provide a prognostic tool that takes into account the treatment regimes that the physician is considering next. For AA, this has been addressed by several prognostic scores [16–18]. However, the scoring system proposed by Chi *et al*. included only patients with AA after docetaxel chemotherapy [16]. Here, six parameters were used to build the model (levels of albumine, LDH, and AP, time from begin of androgen deprivation therapy to start of AA, ECOG, and presence of liver-metastasis). They found that the c-index for the multivariate model was 0.70. In contrast, our model achieved a c-index of 0.74 from the multivariate analysis or 0.80 from analyising the binary-categorized scoring system by considering only four parameters (i.e., ECOG, PSA, LDH and NLR) and was evaluated on patients who underwent docetaxel chemotherapy before or after AA. Therefore our scoring system shows a better capability to prognosticate OS for both treatment situations and offers information on OS probability after 1 und 2 years. However, the prognostic model reported by Chi *et al*. was externally validated in an independent cohort of 286 patients with mCRPC who were sequentially treated in a routine clinical care setting with abiraterone after docetaxel in 11 centers in Canada while our model was only internally validated. In addtion, Chi *et al*. developed their model using a dataset derived from the COU-AA-301 trial with patients who had AA after chemotherapy setting [3].

A trial population is most likely not representative for the patients seen in clinical routine who in a considerable number of cases would never have been enrolled in a randomized controlled trial [24, 25]. This was addressed by Ravi *et al*. who externally validated the prognostic model by Chi *et al*. in an independent cohort of patients treated with abiraterone after docetaxel (*n* = 94) outside a clinical trial and explored its utility in patients treated with abiraterone in the prechemotherapy setting (*n* = 64) [26]. Our cohort was equally derived from consecutive patients seen during clinical routine and we believe that our model is closer to meet the needs of a prognostic tool for treatment planning for AA in real life since our model can prognosticate the suvival probability after 1 and 2 years for both the pre- and the post-chemotherapy situation and it can be applied using only 4 readily available parameters and not 6 parameters.

The prognostic score suggested by Ryan *et al*. comprises data derived from the registration trial for abiraterone in the pre-chemotherapy setting, COU-AA-302 [8]. They identified five risk factors (LDH > upper limit of normal (ULN), number of bone metastases ≥10, PSA >39 ng/ml, presence of lymph node metastasis and hemoglobin ≤ lower limit of normal (LLN)) and the c-index for this model was 0.83, which is marginally better than the c-index of our scoring system [c-index: 0.80; (IQR: 0.79–0.82)]. However, like the previous models, the model introduced by Ryan *et al*. is limited to the pre-chemothearpy setting and evaluated on a data set derived from a RCT. This limits the generation of their models in contrast to our approach which has been build on a real world population and holds true for both pre- and post-chemotherapy settings.

The model proposed by Leibowitz-Amit *et al*. It's close to our approach and partially based on real-life populations from Toronto, Canada (training population) and Sutton, UK (validation cohort). Their model was build based on a data set covering pre- and post-chemotherapy patients. They found that a NLR ≤5 or occurence of bone or lymph node metastases or a combination of both factors seem to be prognostic for 12 months OS and median OS; their prognostic approach is, however, limited to 12 months. In contrast, our scoring system provides useful information about the 2-year survival probability; a low-risk profile determined by 0–2 points is associated with a 2-year survival probability of 50.5%, whereas the high-risk group with 3–4 score shows a survival probability of only 6.4% in the next 2 years.

Given the broad treatment landscape in mCRPC and the dilemma on treatment resistance, it is not only important to choose the next suitable treatment, but also to have a sophisticated treatment strategy that benefits mCRPC patients. We believe, our scoring system is a helpful tool to define a treatment strategy as our system was evaluated on a data set collected consecutively from real-world patients and covering pre- and post-chemotherapy settings for AA. For instance, patients having 3–4 risk factors require a thoughtful treatment sequence to avoid diminishing the life-prolonging effect, while patients with 0–2 are more likly to benefit from AA. Further, our scoring system can be useful for patient counseling.

Despite the rigid analyses, our study is not free from limitations. Although our model was developed using a retrospective dataset that may inherit bias associated with the retrospective nature of our study, our dataset has been prospectively generated. Despite multiple chart reviews, inclusion of some patients without complete data set may introduce selection bias. However, for these patients, existing parameters and survival outcome were in concordance with those of the patients who had a complete dataset. Given the high power and effect size of our approach, we think that the quality of our scoring system was not significantly limited by this fact. Another limitation is that our scoring system was internally validated, but not externally. Finally, despite the fact that our model was rigorously tested and revealed robust results, the prognostic accuracy cannot be perfect, persumably due to the different limitations associated with information gained during the clinical routine. However, using these parameters makes the model very applicable for the real-life clinical condition. Perspectively, we aim to improve our prognostic scoring system with ongoing research by including molecular data for further improvement in the accuracy performance, thereby optimizing or expanding the benefits of sequential treatment strategies for mCRPC patients.

## MATERIALS AND METHODS

### Patients

After ethics committee-approval, we retrospectively reviewed a prospectively maintained database of all patients with mCRPC presenting at the Department of Urology in the Muenster University Medical Center between 12/2009 and 07/2015 to evaluate a prognostic scoring system for patients receiving abiraterone. All patients gave written informed consent before participating. We performed the study according to the declaration of Helsinki. During the reviewed timeframe, a total of 117 patients presented for abiraterone treatment. For some patients (*n* = 28), not all of the parameters for the prognostic model were available. However, for these patients the existing parameters and outcome were consistent with those of the 89 patients for whom the dataset was complete. These patients have one missing parameter on average, which can be imputed using other existing parameters. We therefore decided to include all patients for building the risk score and impute the missing parameters relevant to our study. All patients were diagnosed with mCRPC according to prostate cancer working group-3 (PCWG-3) criteria [27] and received abiraterone either prior to or after chemotherapy (i.e. docetaxel). All men receiving abiraterone prior to docetaxel were asymptomatic or oligo-symptomatic with no need for opiates and in all cases the reported degree of pain no higher than 3 out of 10 on the numeric-rating-scale. Patients treated with abiraterone after docetaxel all had progressive disease on or after chemotherapy. Sixty-nine (59%) men were treated prior to and 48 (41%) after chemotherapy, respectively. Four patients (3.4%) had been treated with Enzalutamide prior to receiving abiraterone (all in post-chemotherapy setting). All patients were on a stable dose of a bone-targeting agent (denosumab or zoledronic acid) at least three months prior to start of abiraterone and during the whole treatment phase or did not receive bone-targeting medication at all.

For all patients, blood was drawn the day before start of abiraterone for baseline analysis of PSA, neutrophils, lymphocytes, LDH, ALP, Calcium and hemoglobin levels. Furthermore, the clinical condition of the patients according to ECOG was assessed for the respective patients.

### Data evaluation

The primary end point used for the model was OS, defined as the time from beginning with AA to date of death of any cause. Ten previously defined prognosticators of OS or baseline clinical parameters were evaluated: age, ECOG performance status, biopsy Gleason score, disease site (defined categorically as lymph node only, bone metastases with no visceral involvement, or any visceral metastases), LDH above ULN, NLR, hemoglobin (Hb), albumin-corrected Calcium (Ca), PSA, and AP. PSA, and AP were highly skewed and the logarithm function was used to transform these variables.

### Development of the score system

The Youden index was applied to determine the optimal cutoffs for continuous variables to predict OS. A penalized Cox’s proportional hazards model using the adaptive least absolute shrinkage and selection operator (LASSO) penalty was used. The main advantage of using penalized methods is that they produce sparse regression coefficients, and the selection of important prognostic factors does not depend on statistical significance. The 95%CI for the adaptive LASSO was derived by adopting the perturbation method. The Akaike information criterion (AIC) was applied to select the model with best goodness of fit and parameters.

For practical reasons and simplicity, the score system was designed by using results from tests widely and easily accessible for physicians and by considering only four parameters that have shown to be associated with survival outcome. The hazard ratio of these parameters should be positively correlated with survival outcome in the univariate analysis to avoid any possible confusion that could occur while scoring these parameters. Because Calcium was inversely associated with outcome, we removed it from our model. Further, the score system consists of binary-categorized parameters; when any of these parameters exceeds the given cut-off, one point is added up to a final score ranging between 0 and 4 points. The final score was then stratified by a median threshold of 2 into low- and high-risk groups.

The score system was evaluated for its discriminative ability in two ways. First, the concordance probability (C-index) was used to measure the discriminative power of the risk score system. Second, the survival prediction for the score system was evaluated using the Kaplan–Meier method. The discrepancy in OS between low-risk and high-risk groups was evaluated based on the log-rank statistic. The survival analysis for the score system was repeated 100 times on a balanced subset with 12 patients that were randomly resampled each time (bootstrapping).

### Statistical analysis

continuous variables, mean, standard deviation, median, and quartiles were presented to show distribution, and the Wilcoxson rank-sum test was used for between-group comparisons. For categorical variables, count and percentage were presented, and χ-square test was used for between-group comparisons. The score system was internally validated by applying 100-times bootstrap resampling. Missing data were imputed using the package ‘missForest’ that applies a random forest trained on the observed values of a data matrix to predict the missing values [28]. All statistical analyses were performed in R, version 3.3.2 (R Development Core Team, Vienna, Austria). The Power and the effect size of contingency table for survival outcome between two groups were calculated using (G*Power, Dusseldorf, Germany) [29].

## CONCLUSIONS

The current study introduces a novel prognostic scoring system for mCRPC patients who are potential candidates for AA treatment. The scoring system is simple and easy to use since it is based on widely available clinical and laboratory parameters. Further, this scoring system offers information on survival probability after one and two years and may help planning sequential treatment regimes within a growing armamentarium of treatment options by for example avoiding AA when the OS prognosis is poor and chemotherapy has not been given before. Athough we performed a rigorous internal validation of the scoring system, a prospective and external validation would support the general application of the proposed scoring system.

## SUPPLEMENTARY MATERIALS



## Abbreviations

PCa: Prostate cancer
mCRPC: metastatic castration resistant prostate cancer
AA: Abiraterone acetate
PSA: prostate-specific antigen
LDH: lactic acid dehydrogenase
AP: alkaline phosphatase
NLR: neutrophil to lymphocyte ratio
RCTs: randomized controlled trials
OS: overall survival
ECOG: Eastern Collaborative Oncology Group Performance Status
CI: Confidence interval
IQR: Interquartile range
ULN: upper limit of normal
LLN: lower limit of normal
PCWG-3: prostate cancer working group-3
Hb: hemoglobin
Ca: albumin-corrected Calcium
LASSO: least absolute shrinkage and selection operator
AIC: Akaike information criterion
C-index: concordance probability
