# Supplementary material for: A prognostic score for overall survival in patients treated with abiraterone in the pre- and post-chemotherapy setting
Source: Oncotarget. 2019 Aug 20;10(49):5082–91. doi: 10.18632/oncotarget.27133 (PMC6707939; doi:10.18632/oncotarget.27133)
Supplement: Supplementary file 1 [file oncotarget-10-5082-s001.pdf]

A prognostic score for overall survival in patients treated with abiraterone in the pre- and post-chemotherapy setting

SUPPLEMENTARY MATERIALS

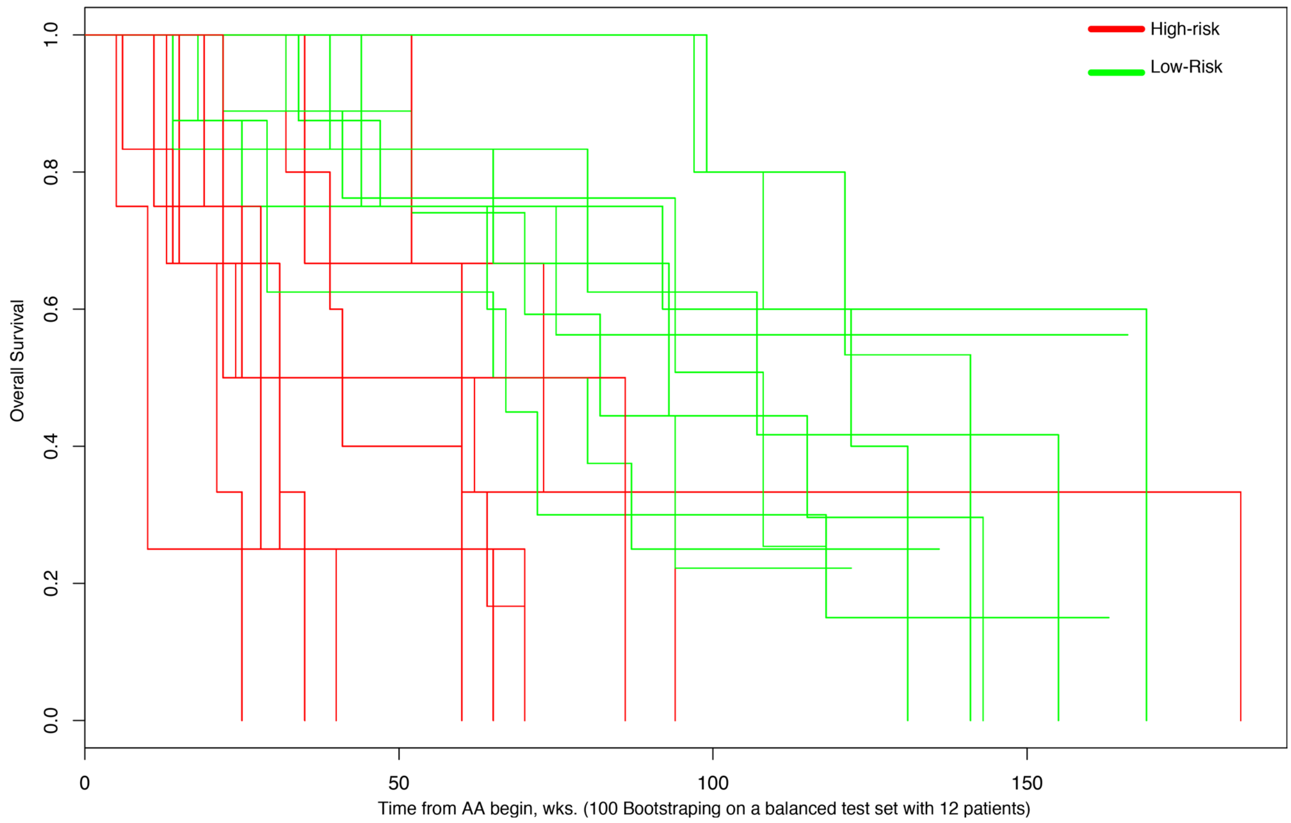

Supplementary Figure 1: Kaplan–Meier Curve of the balanced test set after the 100 bootstrap resampling.
